# Dual-Targeting AKT2 and ERK in cancer stem-like cells in neuroblastoma

**DOI:** 10.18632/oncotarget.27210

**Published:** 2019-09-24

**Authors:** Kwang Woon Kim, Julia Y. Kim, Jingbo Qiao, Rachael A. Clark, Camille M. Powers, Hernan Correa, Dai H. Chung

**Affiliations:** ^1^ Department of Surgery, UT Southwestern Medical Center, Dallas, TX 75390, USA; ^2^ Department of Pediatric Surgery, Vanderbilt University Medical Center, Nashville, TN 37232, USA; ^3^ Department of Pathology, Vanderbilt University Medical Center, Nashville, TN 37232, USA

**Keywords:** chemotherapy, radiotherapy, resistance, AKT2, MAPK

## Abstract

Neuroblastoma remains one of the most difficult pediatric solid tumors to treat. In particular, the refractory and relapsing neuroblastomas are highly heterogeneous with diverse molecular profiles. We previously demonstrated that AKT2 plays critical roles in the regulation of neuroblastoma tumorigenesis. Here we hypothesize that targeting AKT2 could block the signal transduction pathways enhanced in chemo- and/or radiation-resistant neuroblastoma cancer stem-like cells. We found cell proliferation and survival signaling pathways AKT2/mTOR and MAPK were enhanced in cisplatin (CDDP)- and radiation-resistant neuroblastoma cells. Blocking these two pathways with specific inhibitors, CCT128930 (AKT2 inhibitor) and PD98059 (MEK inhibitor) decreased cell proliferation, angiogenesis, and cell migration in these resistant cells. We further demonstrated that the resistant cells had a higher sphere-forming capacity with increased expression of stem cell markers CD133, SOX2, ALDH, Nestin, Oct4, and Nanog. Importantly, the tumorsphere formation, which is a surrogate assay for self-renewal, was sensitive to the inhibitors of AKT2 and MAPK. Taken together, our findings suggest that CDDP- and radiation-resistant cancer stem-like neuroblastoma cells might serve as a useful tool to improve the understanding of molecular mechanisms of therapeutic resistance. This may aid in the development of more effective novel treatment strategies and better clinical outcomes in patients with neuroblastoma.

## INTRODUCTION

Neuroblastoma is an embryonic tumor arising from neural crest cells during early embryogenesis. Neuroblastoma cells sustain the intrinsic capacity of developing embryonic cells to proliferate, vascularize, migrate, and survive at their immature differentiation stages. In patients with high-risk neuroblastoma, 15% have refractory neuroblastoma with no initial treatment response, and more than 50% have relapsed, despite initial remission with treatment.

Therapeutic resistances, such as drug or radiation resistances, are one of the major impediments in clinical therapeutics. Therefore, improving established protocols and developing new strategies for the treatment of relapsed and refractory neuroblastoma are critically necessary. Chemo-resistance is associated with the multi-drug resistance gene *MDR1. MDR1* has been shown to play an important role in the development of drug resistance in malignant tumor cells, functioning as an energy-dependent drug-efflux pump [1]. Increased expression of *MDR1* has been found in some relapsed neuroblastoma after chemotherapy [2]. Radiation resistance is associated with a poor prognosis in cancer patients and represents the main reason for radiotherapy failure, which can ultimately lead to tumor recurrence and metastases [3]. Overcoming radiation-resistance in cancer therapy is an important area of research focus.

Cancer stem cells (CSCs) contain cancer stem cells, tumor-initiating cells or sphere-forming cells. CSCs from a small proportion of tumor cells that have stem cell properties such as: self-renewal capacity, the ability to develop into different lineages, and proliferative potential. The small population of sphere-forming cells in the tumor demonstrate stem-like characteristics and are arrested in a quiescent/dormant state that is resistant to chemotherapy and radiotherapy [4, 5].

Neuroblastoma has a high heterogeneity of cancer stem cells with very different molecular characteristics. Neuroblastoma cells isolated from the bone marrow of high-risk neuroblastoma patients show cancer stem cell properties that are enriched for tumor-initiating capacity [6]. *In vitro* studies in neuroblastoma cell lines have shown that activation of distinct signal transduction pathways can induce neuroblastoma cell differentiation into neuronal [7], chromaffin [8], or Schwannian [9] phenotypes, supporting the existence of cancer stem cells [10].

Recent reports have demonstrated that cancer stem cells are generally resistant to conventional chemotherapy and radiotherapy through activation of cellular pro-survival signaling pathways, PI3K/AKT and MAPK [11, 12]. Neuroblastoma cells, like many cancer cells, have an overactivated AKT/mTOR signaling pathway [13], suggesting involvement in drug- and radiation-resistance mechanisms [11, 14, 15]. Similarly, the mitogen-activated protein kinase (MAPK) also plays an important role in drug resistance and radiation resistance and has been shown to contribute to neuroblastoma drug resistance [16]. However, the MAPK signaling pathway has not been previously found to contribute to radiation resistance. Here we present evidence that activation of the MAPK signaling pathway is enhanced in neuroblastoma radiation resistance. A better understanding of the mechanisms underlying chemo- and radiation-resistances in neuroblastoma cancer stem cells will inevitably lead to novel clinical discoveries in relevant patient populations.

In the current study, we first established cisplatin (CDDP)-resistant and radiation-resistant cell lines by selecting cells under treatment with CDDP and radiation. Then we demonstrated that cancer stem-like properties are present in the drug- and radiation-resistant human neuroblastoma cell lines, BE(2)-C and SK-N-AS. The drug- and radiation-selected resistant cells under the stem cell culture condition also developed spherical cells with cancer stem-like cell properties. This is considered to be a valuable model for the study of CSCs in chemo and radiation resistance in neuroblastoma. In the future, using tumorspheres from selected resistant cells as a pre-clinical xenograft may lead to the discovery of potential molecular mechanisms involved in refractory and relapsing neuroblastoma.

## RESULTS

### Generation of CDDP-resistant and radiation-resistant neuroblastoma cells

*In vitro* drug-resistant and radiation-resistant cell lines have been widely used as a model to study the molecular mechanisms of therapeutic resistance and targeted therapy of drug resistance/radiation resistance in different cancers [1, 17, 18]. These resistant cell lines, which display features of cancer stem cells, are not clearly defined in neuroblastoma. Therefore, we established CDDP- and radiation-resistant human neuroblastoma cell lines using BE(2)-C and SK-N-AS.

**Figure 1:**
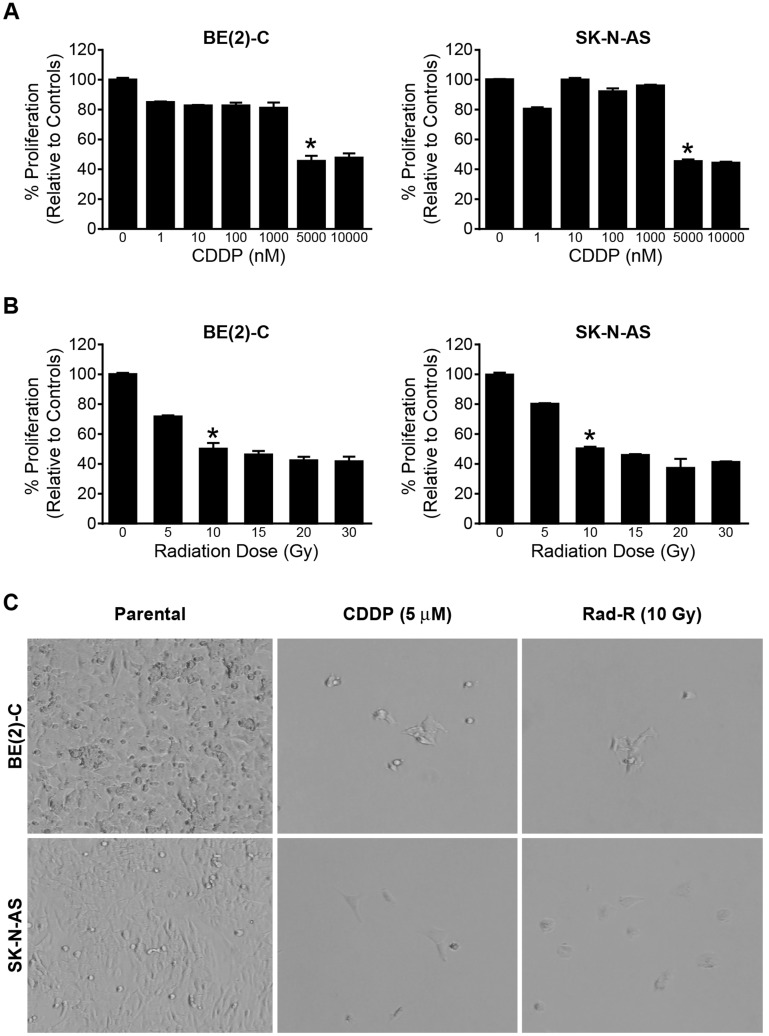
Drug/radiation selected human neuroblastoma cells in a dose-dependent manner. (**A**) BE(2)-C and SK-N-AS cells were treated with increasing concentrations of cisplatin (CDDP) for 96 h. Cell survival was measured using Cell Counting Kit-8 (CCK-8). Data are the mean ± SEM (^*^ = *p* < 0.005). (**B**) BE(2)-C and SK-N-AS cells were irritated with increasing dose of ^137^Cs (5 Gy-30 Gy) for 96 h. Cell survival was determined using CCK-8. Data are the mean ± SEM (^*^ = *p* < 0.005). (**C**) Drug/radiation surviving cells were generated after 7 days in the BE(2)-C and SK-N-AS cells treated with 5 μM of CDDP and irradiated with 10 Gy of ^137^Cs respectively. Representative photographs are shown for surviving cells after 7 days treatment of CDDP and ^137^Cs.

To establish a drug resistant cell line, we first determined the dose at which 50% of cell survival was inhibited. A concentration of 5000/well was used to plate the cells in 96-well plates. These were cultured for 96 h with the addition of cisplatin at variant dosages. The cell survival was then measured with Cell Counting Kit-8 (CCK-8). We found that 5 μM of CDDP lead to 50% of cell survival inhibition for both cell lines (Figure 1A). In addition, we also found the IC_50_ of cisplatin to be 2.285 μM and 3.203 μM in the BE(2)-C and SK-N-AS cell lines, respectively (Supplementary Figure 1A).

In order to establish a radiation-resistant cell line, the radiation dose at which 50% of cell survival inhibition was evaluated. Both cell lines were irradiated with ^137^Cs irradiator (J. L. Shepherd and Associates, Glendale, CA, USA) at room temperature (dose rate 1.8 Gy/min). The dose of radiation at which 50% of survival was inhibited in both cell lines was 10 Gy (Figure 1B and Supplementary Figure 1B).

We then used the dose of cisplatin and radiation at which 50% of cell survival was inhibited to successfully establish four resistant cell lines: CDDP-resistant cell lines, CDDP-R BE(2)-C and CDDP-R SK-N-AS, and radiation-resistant cell lines, Rad-R BE(2)-C and Rad-R SK-N-AS. The two cell lines were treated with 5 μM of cisplatin and 10 Gy of radiation and then plated at 3 × 10^5^ cells/dish into 100 mm × 20 mm plates for seven days. After seven days, 5% of the total cells (3 × 10^5^ cells) survived. These were allowed to recover for additional 25 days, as shown in Figure 1C. This development period was carried out for approximately 6 months. At six months, the cell lines were treated with 5 μM of CDDP and 10 Gy of radiation to confirm resistance in each cell line.

### Proliferation capacity in CDDP-R/Rad-R neuroblastoma cells

**Figure 2:**
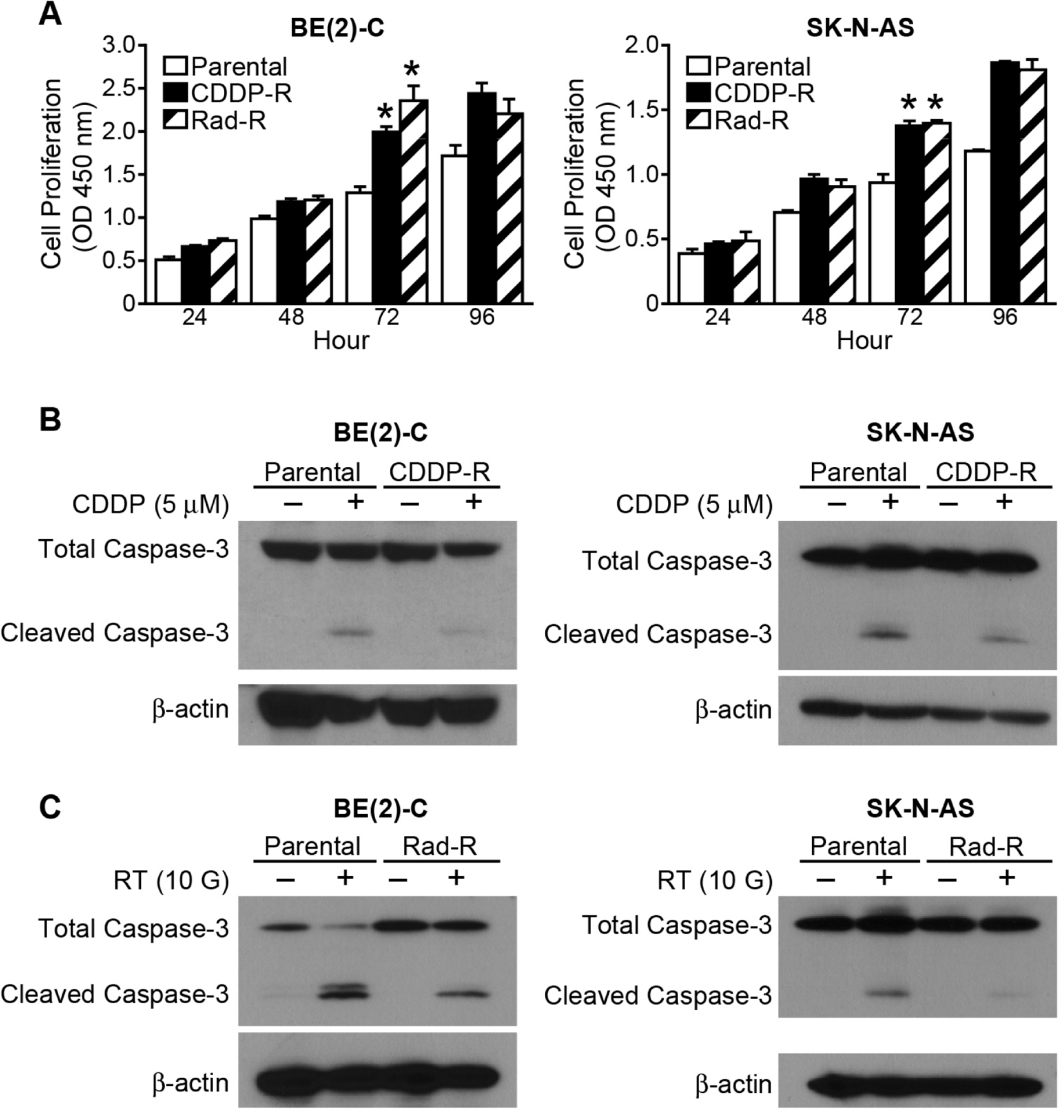
CDDP-R/Rad-R human neuroblastoma cells increase proliferation and inhibit programmed cell death. (**A**) CDDP-R/Rad-R BE(2)-C cells and CDDP-R/Rad-R SK-N-AS cells demonstrated increased proliferation when compared with parental BE(2)-C cells and SK-N-AS cells. Data are the mean ± SEM. ^*^ Significantly different at *p* < 0.005 vs. Parental. (**B**) Cleaved caspased-3 was inhibited in the CDDP-R BE(2)-C cells and CDDP-R SK-N-AS cells when compared with parental BE(2)-C cells and SK-N-AS cells after treatment with CDDP (5 μM) for 48 h. (**C**) Cleaved caspased-3 was inhibited in the Rad-R BE(2)-C cells and Rad-R SK-N-AS cells when compared with parental BE(2)-C cells and SK-N-AS cells after irritation with ^137^Cs (10 Gy) for 48 h. β-actin served as a protein loading control.

In order to characterize these resistant cells we performed cell proliferation assays of the resistant cells and their respective parent cells. The cells were cultured up to 96 h, and the resistant cells showed significantly increased cell proliferation after only 72 h in all four resistant cell lines, CDDP-R/Rad-R BE(2)-C and SK-N-AS cells, when compared to parent cells (Figure 2A). In addition, we studied the programmed cell death of the cell lines to confirm persistent resistance in resistant cell lines to CDDP and radiation. The resistant cells and the parent cells were treated with CDDP (5 μM) and radiation (10 Gy). The resistant cells showed decreased levels of cleaved caspase-3 expression when compared to their respective parental cells after treatment (Figure 2B and 2C). However, our data showed that combination treatment with CCT128930 (10 uM), PD98059 (100 uM), and CDDP (5 μM) induced more cleaved caspase-3 or less total caspase-3 than CDDP (5 μM) treatment in the resistant cells (Supplementary Figure 2A and 2B).

To further confirm CDDP-R BE(2)-C and CDDP-R SK-N-AS cells, we tested the previously calculated IC_50_ doses on both the parental cell and resistant cells. The CDDP-R BE(2)-C and CDDP-R SK-N-AS displayed an increased IC_50_ dose compared to parent cells, consistent with CDDP resistance (Supplementary Figure 3).

Taken together, these results demonstrate that the resistant cells spontaneously showed increased growth ability and persistent resistance.

### Identification of increased GRP-R/AKT2 and MAPK signaling pathways in CDDP-R/Rad-R neuroblastoma cells

**Figure 3:**
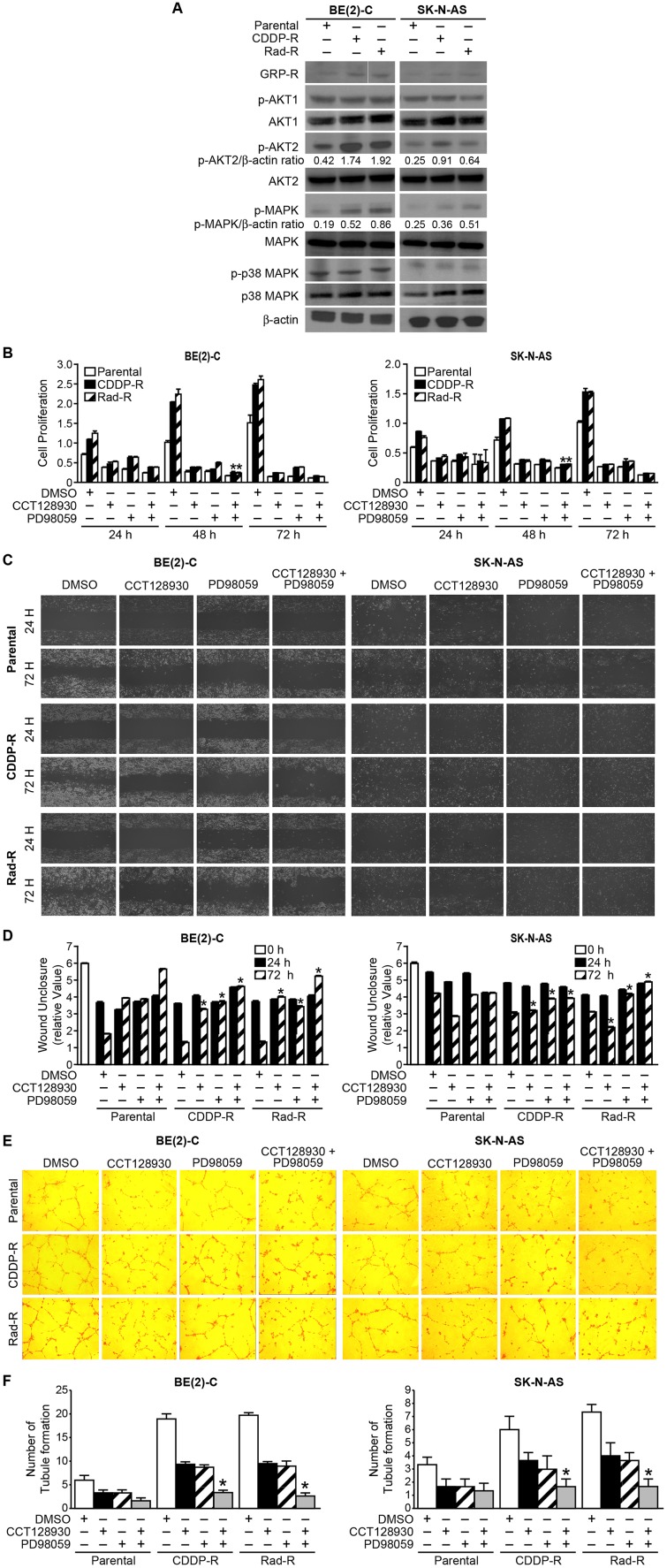
Activation of GRP-R/AKT2 and MAPK signaling pathways in CDDP-R/Rad-R BE(2)-C cells and CDDP-R/Rad-R SK-N-AS cells. (**A**) Levels of GRP-R/p-AK2 and p-MAPK were spontaneously increased in CDDP-R/Rad-R BE(2)-C cells and CDDP-R/Rad-R SK-N-AS cells. β-actin protein was used as a loading control. (**B**) Parental BE(2)-C cells, CDDP-R/Rad-R BE(2)-C cells, parental SK-N-AS cells, and CDDP-R/Rad-R SK-N-AS cells were treated with DMSO, CCT128930 (AKT2 selective inhibitor, 10 μM), PD98059 (MAPK inhibitor, 100 μM), and combined CCT128930/PD98059 over a time course, and cell proliferation was measured using Cell Counting Kit-8. Data are the mean ± SEM. ^*^ Significantly different at *p* < 0.005 vs. parental. (**C**) Parental BE(2)-C cells, CDDP-R/Rad-R BE(2)-C cells, parental SK-N-AS cells and CDDP-R/Rad-R SK-N-AS cells were treated with DMSO, CCT128930 (AKT2 selective inhibitor, 10 μM), PD98059 (MAPK inhibitor, 100 μM), and combined CCT128930/PD98059. After 24 h, scratches were made using 200 μl tips. Wound closure was measured from microscopic images at 24 h and 72 h after wounding (100× magnification). (**D**) Data are representative of the mean distance of unclosure from three independent experiments (^*^ = *p* < 0.05 vs. DMSO). (**E**) HUVECs were plated on 24-well plates coated with Matrigel and incubated with cell culture media from parental BE(2)-C cells, CDDP-R/Rad-R BE(2)-C cells, parental SK-N-AS cells and CDDP-R/Rad-R SK-N-AS cells treated with DMSO, CCT128930 (AKT2 selective inhibitor, 10 μM), PD98059 (MAPK inhibitor, 100 μM), and combined CCT128930/PD98059 for 48 h. Tubule staining was performed in triplicate and representative images shown (×200). (**F**) The numbers of tubule were counted. Values shown are mean ± SEM of three separate experiments (^*^ = *p* < 0.05 vs. DMSO).

We have previously reported that gastrin-releasing peptide receptor (GRP-R) mediated tumor progression in neuroblastoma occurs via activation of the PI3K/AKT pathway [19]. In particular, we reported that AKT2 was critical in the regulation of neuroblastoma tumorigenesis [20]. However, the exact role of GRP-R/AKT2 in CDDP-R/Rad-R neuroblastoma cells has not been studied yet. Here, we examined the GRP-R expression and activation of AKT2 in the CDDP-R/Rad-R BE(2)-C and SK-N-AS cells. The resistant cell lines clearly showed increased GRP-R expression levels as well as increased AKT2 activation (Figure 3A). However, neither increased AKT1 activation nor increased total AKT1 protein level was observed (Figure 3A).

As shown in Figure 2A, we found that the resistant cells demonstrated increased spontaneous cell proliferation. MAPK and p38 MAPK are associated with increased cell proliferation [21]. In order to study cell proliferation in resistant cells, MAPK and p38 MAPK activation was measured (Figure 3A). We found that the resistant cells showed increased MAPK activation when compared to parent cells, but not p38 MAPK activation. However, the total protein levels of AKT2, MAPK and p38 MAPK did not change in the resistant cells. These results showed that the resistant cells are associated with both GRP-R/AKT2 and MAPK pathways, indicating that these survival factors may transform resistant cells to cancer stem-like cell phenotypes.

To further evaluate AKT2 and MAPK in resistant cells, the selective AKT2 inhibitor, CCT128930 and the MAPK inhibitor, PD98059 were used. We performed proliferation assays on both parental and resistant cells treated with DMSO, CCT128930 (10 μM), PD98059 (100 μM), or both CCT128930 and PD9805 for 24, 48, and 72 h. As shown in Figure 3B, we found that CCT128930 and PD98059 inhibited CDDP-R BE(2)-C cell proliferation by 5.3 fold and 6 fold at 48 h, respectively. CCT128930 and PD98059 inhibited Rad-R BE(2)-C cell proliferation by 5.8 fold and 4.5 fold at 48 h, respectively. In particular, the combination treatment group, with both CCT128930 and PD98059, significantly inhibited proliferation by 8.1 fold for CDDP-R BE(2)-C cells and 9.3 fold for Rad-R BE(2)-C cells (Figure 3B, left panel). Similarly, we observed that both CCT128930 and PD98059 inhibited CDDP-R SK-N-AS cell proliferation by 2.8 fold and Rad-R SK-N-AS proliferation by 3.8 fold at 48 h. The combination treatment group of both CCT128930 and PD98059 also significantly inhibited proliferation by 3.5 fold for both the CDDP-R and Rad-R SK-N-AS cells (Figure 3B, right panel).

In addition, we wanted to examine whether combination treatments of CCT128930 and PD98059 with CDDP, or CCT128930 and PD98059 with radiation inhibited proliferation in CDDP-R/Rad-R BE(2)-C cells and CDDP-R/Rad-R SK-N-AS cells when compared to CDDP or radiation alone. Our data showed that CDDP-R/Rad-R BE(2)-C cells and CDDP-R/Rad-R SK-N-AS cells treated with CDDP or radiation alone had sustained cell proliferation, however, cells treated with a combination of CCT128930, PD98059, and CDDP or radiation, CCT128930, and PD98059 had significantly decreased cell proliferation (Supplementary Figure 2C and 2D).

We next determined whether targeting AKT2 and MAPK affected cell migration in CDDP-R/Rad-R BE(2)-C and CDDP-R/Rad-R SK-N-AS cells. We performed wound healing assays at 24 and 72 h. Wound unclosure was measured by microscopy, and relative values were calculated. We found that CDDP-R/Rad-R BE(2)-C and CDDP-R/Rad-R SK-N-AS cells spontaneously increased cell migration at 72 h after wounding when compared to parental cells, suggesting that these resistant cells may have metastatic characterization (Figure 3C). We also investigated whether CDDP-R/Rad-R BE(2)-C and CDDP-R/Rad-R SK-N-AS cells treated with CCT128930 (10 μM), PD98059 (100 μM), or combination of CCT128930 and PD98059 had decreased cell migration similar to parental cells at 72 h. As expected, we observed that CCT128930, PD98059, or combination of CCT128930 and PD98059 significantly reduced cell migration in the resistant cells (Figure 3D).

In addition, to examine whether DMSO, CCT128930, PD98059, or both CCT128930 and PD98059 affected tubule formation and, in turn, angiogenesis of CDDP-R/Rad-R BE(2)-C and CDDP-R/Rad-R SK-N-AS cells *in vitro*, we utilized human umbilical vein endothelial cells (HUVECs). Cell culture supernatant from parental, CDDP-R/Rad-R BE(2)-C and CDDP-R/Rad-R SK-N-AS cells were used for HUVECs (Figure 3E). We found that the number of tubules formed by HUVECs grown in media from CDDP-R/Rad-R BE(2)-C and CDDP-R/Rad-R SK-N-AS cells was significantly increased when compared to parental cells, suggesting an increase in VEGF secretion in resistant cells, and we confirmed that VEGF secretion was 4.2/4.1- and 2.9/3-fold increase in CDDP-R/Rad-R BE(2)-C and CDDP-R/Rad-R SK-N-AS cells respectively (Supplementary Figure 4). However, as expected, cells treated with CCT128930, PD98059, or both CCT128930 and PD98059 had decreased tubule formation in both HUVECs grown in media from CDDP-R/Rad-R BE(2)-C and CDDP-R/Rad-R SK-N-AS cells when compared with vehicle DMSO, suggesting that the resistant cells are involved with angiogenesis (Figure 3F). Taken together, our data show that CDDP-R/Rad-R BE(2)-C and CDDP-R/Rad-R SK-N-AS cells demonstrate characterization associated with cancer stem cells.

### Identification of cancer stem cell markers in CDDP-R/Rad-R BE(2)-C and SK-N-AS cells

**Figure 4:**
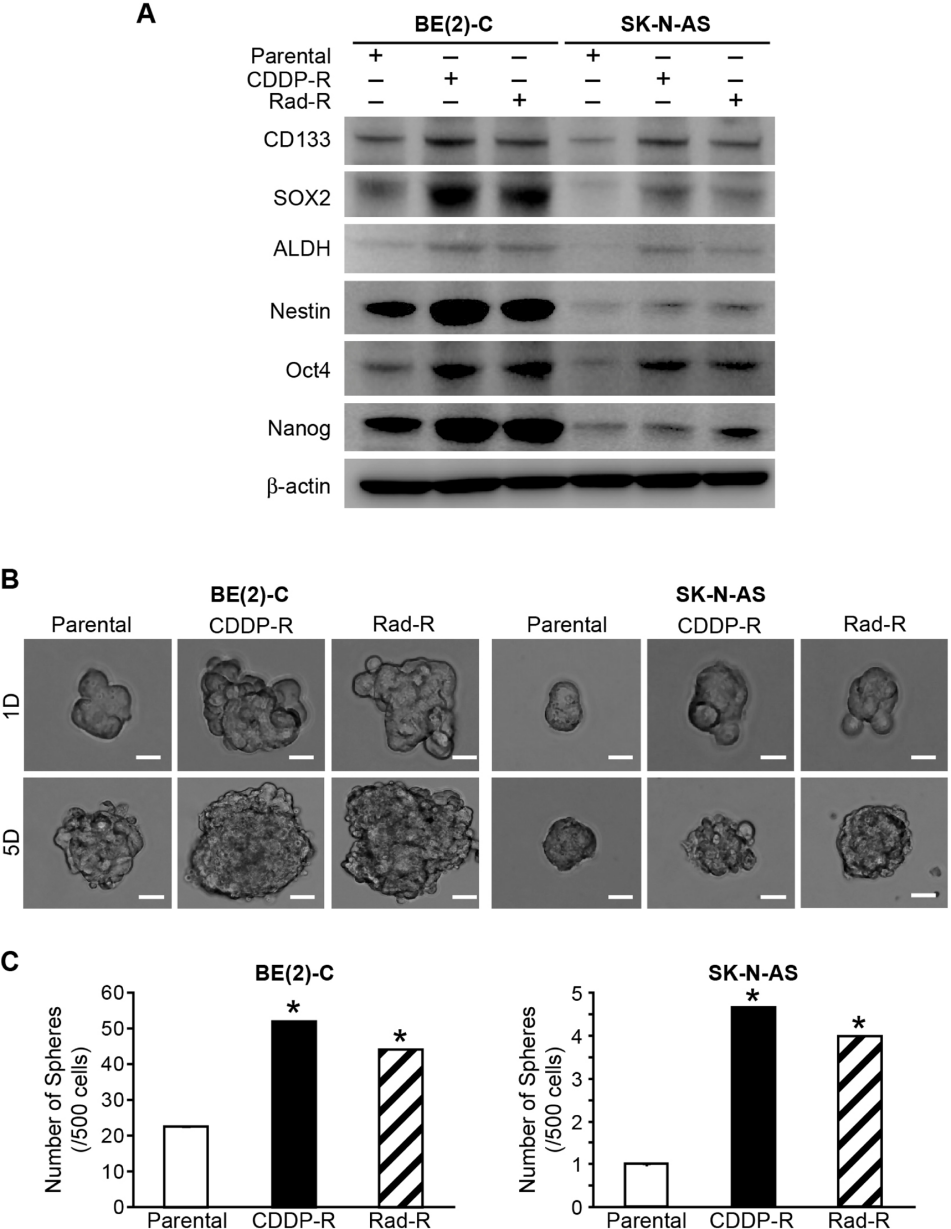
Markers of cancer stem cells in drug/radiation selected cells. (**A**) Parental BE(2)-C cells, CDDP-R/Rad-R BE(2)-C cells, parental SK-N-AS cells, and CDDP-R/Rad-R SK-N-AS cells demonstrated increased expression of several stem cell markers, CD133, SOX2, ALDH, Nestin, Oct4, and Nanog, by Western blotting. β-actin served as a protein loading control. (**B**) Parental BE(2)-C cells, CDDP-R/Rad-R BE(2)-C cells, parental SK-N-AS cells, and CDDP-R/Rad-R SK-N-AS cells demonstrated neurosphere formation at 1 day and 5 days. Representative photographs are shown for floating spheres (scale bar = 100 μm). (**C**) CDDP-R/Rad-R BE(2)-C cells and CDDP-R/Rad-R SK-N-AS cells were altered to neurosphere formation. Spheres (a diameter >100 μm) were counted using inverted microscopy and quantified. Values shown are mean ± SEM of three separate experiments (^*^ = *p* < 0.05 vs. Parental).

In order to further explore the stem-cell like characterizations of CDDP-R/Rad-R BE(2)-C and CDDP-R/Rad-R SK-N-AS cells, we studied the expression of cancer stem cell markers. First, we compared the protein levels of CD133, SOX2, ALDH, Nestin, Oct4, and Nanog in parental cells, CDDP-R/Rad-R BE(2)-C and SK-N-AS cells by Western blotting. Expressions of cancer stem cell markers CD133, SOX2, ALDH, Nestin, Oct4, and Nanog were enhanced in both CDDP-R/Rad-R BE(2)-C and SK-N-AS cells in comparison to parental cells. These data suggest that our CDDP-R/Rad-R BE(2)-C and SK-N-AS cells demonstrate characteristics of cancer stem-like cells (Figure 4A).

We then sought to further characterize the CDDP-R/Rad-R BE(2)-C and SK-N-AS cells *in vitro*. The cell lines were cultured in serum-free neurobasal media and ultra-low attachment culture dishes that permit the formation of neurospheres. Neuroblastoma derived neurospheres have enriched cancer stem cells and exhibit stem cell-like properties that are involved in the failure to achieve long-term remission in neuroblastoma. Neurospheres favor the expression of neural stemness markers and are enriching for tumor-initiating cells [22, 23]. The neurospheres were grown in ultra-low attachment plates using serum-free media, and sphere formation was quantified at 1 day and 5 days. Our results showed that CDDP-R/Rad-R BE(2)-C and SK-N-AS cells has significantly increased sphere size (Figure 4B) when compared with parental cells. Particularly, we observed that CDDP-R and Rad-R BE(2)-C cells had a 2.3-and 2-fold increase in the number of spheres compared to parental cells, respectively. In addition, CDDP-R/Rad-R SK-N-AS cells had a 4.7 fold and 4 fold, respectively, increase in the number of spheres compared to parental cells.

**Figure 5:**
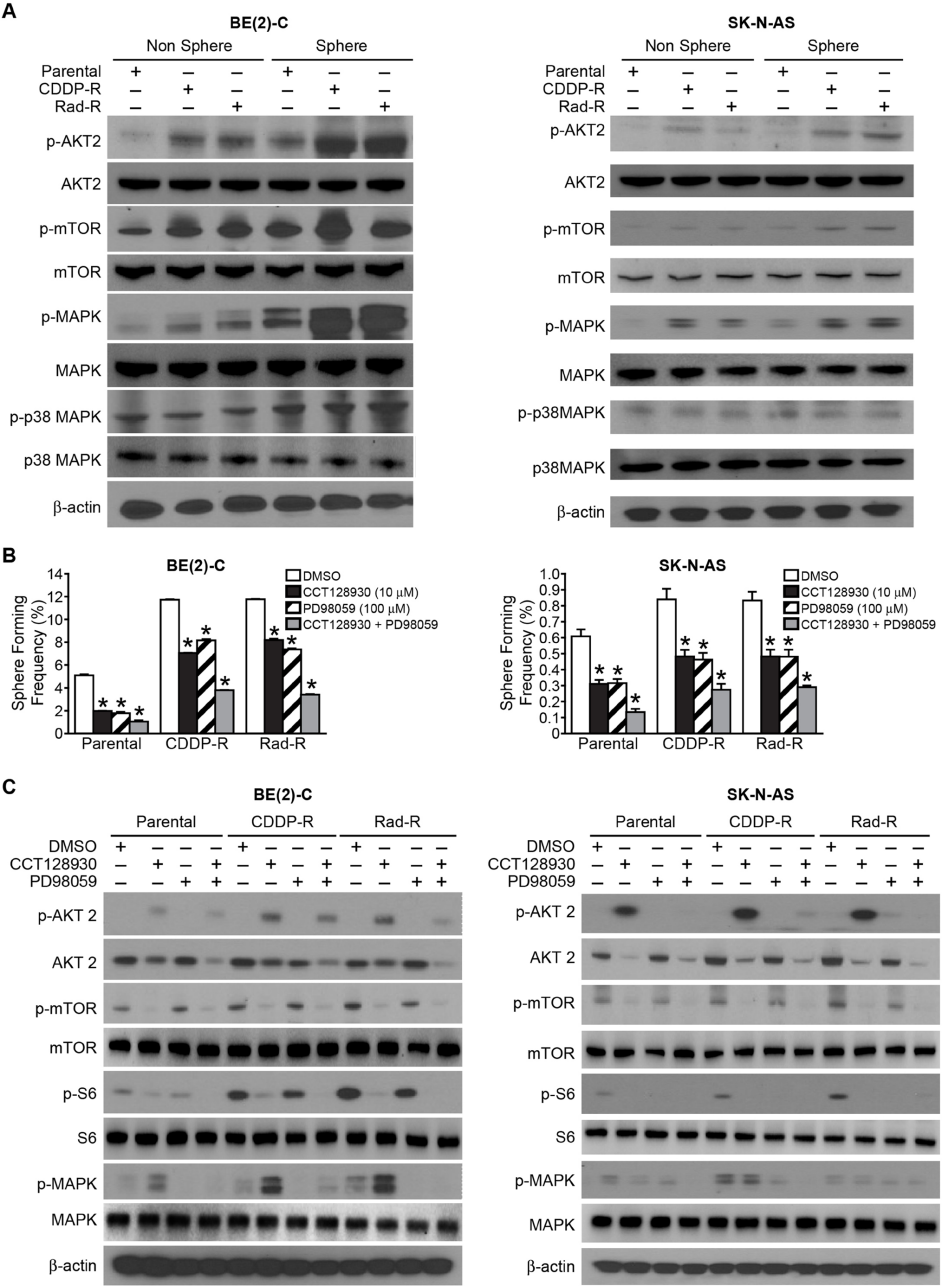
Signaling pathways in cancer stem-like cells with activated AKT2/mTOR and MAPK in Drug/Radiation selected cells. (**A**) Sphere formation selected for subpopulations of CDDP-R/Rad-R BE(2)-C cells and CDDP-R/Rad-R SK-N-AS cells. The neurospheres demonstrated activation of p-AKT2, p-mTOR, p-MAPK, not p-p38 MAPK by Western blotting. β-actin served as a protein loading control. (**B**) Parental BE(2)-C cells, CDDP-R/Rad-R BE(2)-C cells, parental SK-N-AS cells and CDDP-R/Rad-R SK-N-AS cells were cultured in sphere formation media with DMSO, CCT128930 (AKT2 selective inhibitor, 10 μM), PD98059 (MAPK inhibitor, 100 μM), and combined CCT128930/PD98059 and a limiting dilution analysis was performed. Floating spheres were observed using inverted microscopy. Spheres with a diameter > 100 um were counted and quantified. Data represent frequency of sphere-formation (%) determined by 8-dose dilution and 6 wells/group in DMSO and treatment groups (mean ± SEM; ^*^ = *p* < 0.05 vs. DMSO). (**C**) Sphere formed from subpopulations of parental BE(2)-C cells, CDDP-R/Rad-R BE(2)-C cells, parental SK-N-AS cells, and CDDP-R/Rad-R SK-N-AS cells demonstrated deactivated levels of p-AKT2/p-mTOR/P-S6R protein and p-MAPK with DMSO, CCT128930 (AKT-2 selective inhibitor, 10 μM), PD98059 (MAPK inhibitor, 100 μM), and combined CCT128930/PD98059 treatments by Western blotting for 48 h. β-actin served as a protein loading control.

As shown in Figure 2, our resistant cells resulted in increased cell proliferation as well as a resistant apoptosis when compared to parental cells, implying that molecular signaling pathways related to cell survival were activated in our resistant cells. Therefore, we wanted to investigate whether AKT2, mTOR, MAPK, and p38 MAPK were activated in the spheres. As expected, we observed that AKT2, mTOR, and MAPK were spontaneously activated in our resistant cells compared to parental cells. However, total AKT2, total mTOR, and total MAPK protein level were not increased in the resistant cells. Interestingly, the p38 MAPK pathway was not activated in CDDP-R/ Rad-R BE(2)-C and SK-N-AS cells in comparison with parental cells (Figure 5A).

We also wanted to examine whether these molecules were activated in CDDP-R/Rad-R BE(2)-C and SK-N-AS cells cultured in ultra-low attachment culture dishes with serum-free neurobasal media. Our results showed that AKT2, mTOR, and MAPK were spontaneously activated and enhanced in spheres. P38 MAPK was not activated in either the resistant cells or parental cells (Figure 5A). We observed that the total protein levels of AKT2, mTOR, MAPK, and p38 MAPK were not enhanced in spherical cells. These results demonstrate that our resistant cells are potentially altered to display a neurosphere cancer stem-like cell phenotype.

To better quantify the effects of the AKT2 pathway, which is upstream of mTOR, and the MAPK pathway in sphere-forming frequency (SFF), we performed a limiting dilution analysis to assess the SFF of parent BE(2)-C, CDDP-R/Rad-R BE(2)-C, parent SK-N-AS, and CDDP-R/Rad-R SK-N-AS cell lines by using the selective AKT2 inhibitor, CCT128930, and MAPK inhibitor, PD98059. Ninety-six-well plates were scored for sphere formation over 4 days of incubation. At first, we observed that CDDP-R/Rad-R BE(2)-C cells displayed a 2.2-fold and 2.1-fold increase in SFF respectively. When compared with parental cells, CDDP-R/Rad-R SK-N-AS cells had a 1.4-fold and 1.3-fold increase in respective SFF. Treatment with CCT 128930 (10 μM) and PD98059 (100 μM) reduced the SFF of BE(2)-C and SK-N-AS parental cells by 2.5 and 2 fold, respectively. The combination group of CCT128930 and PD98059 resulted in a 3-fold decrease in SFF of both BE(2)-C and SK-N-AS parental cells (Figure 5B). In particular, as expected, we observed that treatment with CCT128930 and PD98059 in CDDP-R/Rad-R BE(2)-C and CDDP-R/Rad-R SK-N-AS cells induced a 2-fold decrease in SFF and the combination group led to a 3-fold decrease in SFF (Figure 5B). Taken together, our findings demonstrate that sphere formation of CDDP-R/Rad-R BE(2)-C and SK-N-AS can be decreased by inhibiting both AKT2/mTOR and MAPK pathways.

**Figure 6:**
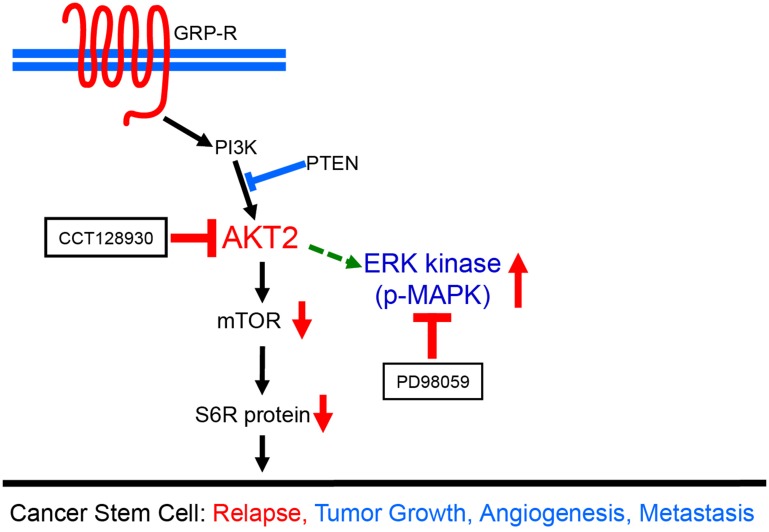
A schematic diagram demonstrating dual targeting of AKT2 and MAPK pathways in neuroblastoma cancer stem cells. AKT2 and MAPK pathways were spontaneously overactivated in CDDP-R/Rad-R BE(2)-C cells and CDDP-R/Rad-R SK-N-AS cells growing up in neurosphere media. Despite targeting AKT2 by CCT128930, the MAPK pathway is still overactivated in neurosphere formation due to the effect of AKT2 inhibition. Therefore, dual-targeting of AKT2 and MAPK is far more effective in neuroblastoma cancer stem-like cells. Our data demonstrated that dual targeting of AKT2 and MAPK, by CCT128930 and PD98059 respectively, provides more effective therapeutic strategies for neuroblastoma cancer stem cells which can initiate a risk of relapse.

To confirm whether the sphere-forming capacity of CDDP-R/Rad-R BE(2)-C and SK-N-AS cells was inhibited via AKT2/mTOR and MAPK pathways, we measured AKT2, mTOR, S6R protein and MAPK by Western blotting (Figure 5C). CDDP-R/Rad-R BE(2)-C and SK-N-AS cells treated with the combination treatment groups of both CCT128930 and PD98059 were cultured with serum-free neurobasal media and demonstrated decreased levels of p-AKT2, total AKT2, p-mTOR, p-S6R protein, and p-MAPK similar to parental cells. As seen previously, the total mTOR, total S6R protein, and total MAPK protein levels did not change. These results demonstrated that activation of the AKT2/mTOR or MAPK pathways may be related to sphere formation in drug and radiation-resistant neuroblastoma cells (Figure 6).

## DISCUSSION

Establishing resistant cancer cell lines has become a clinically relevant *in vitro* model to study therapeutic resistance and disease relapse in cancer patients. Many different drug-/radiation-resistant cell lines have been selected with various strategies from commercial cell lines or patient-derived cell lines in various cancers [17]. In neuroblastoma, therapeutic drug/radiation resistance induced during the clinical courses is one of the major impediments to the treatment of patients with advanced-stage, high-risk neuroblastoma. In particular, many different mechanisms of drug resistance have been found, such as the enhanced expression of anti-apoptosis gene Bcl-2 [24], which amplifies neural apoptosis inhibitory protein (NAIP), after treatment with retinoic acid [25].

In this study, we established *in vitro* CCDP-resistant and radiation-resistant cell lines with two well-established human neuroblastoma cell lines, BE(2)-C, *MYCN* amplified; and SK-N-AS, *MYCN* non-amplified. The aim of this study was to determine the molecular mechanisms and roles of the PI3K/AKT and MAPK pathways on the establishment of cancer stem-like cells in a population of CDDP-R/Rad-R BE(2)-C and SK-N-AS cells. It has been reported that oncogenic resistance is associated with highly aggressive cancer phenotypes with rapid cell proliferation and modulates therapeutic-induced apoptosis resistance [26, 27]. Consistent with previous reports, our study found that only 5% of cells survived (3 × 10^5^ cells) when treated with the 50% survival doses of CDDP (5 μM) and radiation (10 Gy) for 7 days. In addition, under retreatment with 5 μM of CDDP and 10 Gy of radiation, the resistant cells demonstrated a decrease in active caspase 3, compared to their respective parental cells. This indicates that the selected cells dramatically increased cell proliferation and induced resistance to programmed cell death, supporting the existence of cancer stem-like cell capability.

Dysregulation of various signaling pathways have been found in cancers, including PI3K/AKT, MAPK, WNT/β-catenin, and TGF-β/Smad. These pathways play a role in regulating tumor initiation, tumor growth, cells senescence, cell death, differentiation, and metastasis in different stages of cancer. The enhanced mitogenic signaling pathways of PI3K/AKT and RAS/MAPK result in sustained chronic proliferation in cancer cells. We previously found that the PI3K/AKT signaling pathway is quite active in high-risk neuroblastoma and is regulated by mitogen gastrin-releasing peptide through its G protein-coupled receptor [26, 28]. Notably, AKT activation has been shown to be an indicator of malignancy and chemo-resistance in neuroblastoma [29, 30]. Furthermore, we have previously demonstrated that AKT2 plays a key role in regulating tumor metastasis in neuroblastoma [20]. In the present study, we found that AKT2 activity was enhanced in resistant cells and that treatment with an AKT2 specific inhibitor, CCT128930, could various inhibit oncogenic phenotypes such as cell proliferation, cell anchorage-independent growth, angiogenesis, and cell migration of the CDDP-R/Rad-R BE(2)-C and SK-N-AS cells.

A recent study reported that 78% of relapsed tumors showed activating mutations in the RAS-MAPK pathway [31]. In addition, our results showed that the activity of MAPK pathway was enhanced in CDDP-R/Rad-R BE(2)-C and SK-N-AS cells, and that they were sensitive to a resistant phenotype through the treatment with a MAPK inhibitor, PD98059. In particular, the combination treatment, using two inhibitors of AKT2 and MAPK, dramatically enhanced synergic sensitivity to chemotherapy in CDDP-R/Rad-R BE(2)-C and SK-N-AS cells. Although we found that AKT2 and MAPK were activated in the established resistant cells, p38 MAPK was not activated in the resistant cells or parental cells. Our results are consistent with the fact that activated p38 MAPK does not contribute to the acquisition and maintenance of cancer stem cell properties in non-small cell lung cancer (NSCLC) cells [32]. Taken together, these results demonstrate cancer stem-like properties in the established resistant cell lines and will lead to the investigation of additional cancer stem-like properties of CDDP-R/Rad-R BE(2)-C cells and CDDP-R/Rad-R SK-N-AS cells.

Cancer stem cells, a small population of tumor cells, are considered to be the source of drug resistance and radiation resistance [4, 12]. These cells are generally quiescent in G0 phase and have self-renewal capacity. CSCs have been thought to be involved in cancer initiation, maintenance, metastasis, and recurrence. The development of novel therapeutic systems targeting cancer stem cells have been carried out [33]. Pediatric cancers, including neuroblastoma, are embryonic tumors, arising from cellular populations that have not completed the process of terminal differentiation during fetal and/or postnatal development [34]. Here, we demonstrated that stem cell markers such as CD133, SOX2, ALDH, Nestin, Oct4, and Nanog were enhanced in CDDP-R/Rad-R BE(2)-C cells and CDDP-R/Rad-R SK-N-AS cells. Among these stem cell markers, anti-CD133 therapy has been tested and resulted long-term disease-free tumor survivors, suggesting that anti-CD133 therapy might be associated with drug delivery, forming antibody-drug conjugates that enhance the effect on CD133+ cancer stem cells and increases elimination of CSCs [33]. Further, we characterized the resistant cells *in vitro* by culturing them in serum-free neurobasal media. We found that neuroblastomas cell-derived neurospheres were dramatically increased in these resistant cells. Our results are consistent with previous papers that showed drug- resistance and radiation-resistant cell lines demonstrate stem–like properties [35, 36].

Cancer stem cells are associated with the activation of survival signaling pathways, such as PI3K/AKT and MAPK, that aid in resistance to chemo- or radiation therapies [11, 12]. Consistent with these previous reports, our results showed that, compared to parental cells, AKT2, mTOR, and MAPK were spontaneously activated in CDDP-R/Rad-R BE(2)-C cells and CDDP-R/Rad-R SK-N-AS cells when cultured in ultra-low attachment culture dishes with serum-free neurobasal media. In both parent and resistant cell lines p38 MAPK was not activated. We found that sphere-forming frequency, which is a surrogate assay for self-renewal, was enhanced in resistant cells compared to parental cells that did not have activation the AKT2 MAPK pathways. This further supports the existence of cancer stem-like cell function, via both AKT2/mTOR pathway and MAPK pathway, in our resistant cell lines. Therefore, the residual population of chemotherapy-resistant and radiotherapy-resistant tumor cells are thought to be cancer stem-like cells, capable of regenerating tumor relapse.

In conclusion, we demonstrated that after selection with CDDP and radiation, the CDDP-R/Rad-R BE(2)-C cells and CDDP-R/Rad-R SK-N-AS cells have enhanced cancer stem cell characteristics, leading to tumor initiation, progression and relapse. From these findings, we can develop an understanding of the molecular mechanisms, like the AKT2 or MAPK pathways, of chemo- and radiation-resistant cancer cells with stem cell-like phenotypes. Understanding the underlying mechanisms is key to developing strategies to overcome therapeutic resistance in patients with relapsed and refractory neuroblastoma.

## MATERIALS AND METHODS

### Cell culture, reagents, and antibodies

Neuroblastoma cells BE(2)-C and SK-N-AS were cultured in RPMI 1640 (Mediatech, Cat No. 10-040-CV) supplemented with 10% fetal bovine serum (Sigma-Aldrich, Cat No. F2442) at 37° C and humidified 5% CO_2_. HUVECs obtained from Dr. M. Freeman (Vanderbilt University Medical Center) were cultured in EMM-2 supplemented with Growth Factors (EGM-2 SingleQuot kit, Lonza, Cat No. CC-4176) at 37° C and humidified 5% CO_2_. Cisplatin (cis-diamminedichloroplatinum; CDDP) was purchased from Sigma-Aldrich (Cat No 15663-27-1), CCT128930, AKT2 selective inhibitor, was purchased from Selleckchem (Cat No. 885499-61-6), and PD98059, MEK inhibitor, was purchased from Adooq Bioscience LLC (Cat No. A10705-100). Primary antibodies used include GRP-R (1:1000, ab39963, Abcam), Caspase-3 (1:1000, Cat No 9996, Cell Signaling), p-AKT1 (1:1000, Cat No 9018, Cell Signaling), AKT1 (1:1000, Cat No 9272, Cell Signaling), p-AKT2 (1:1000, Cat No 8599, Cell Signaling), AKT2 (1:1000, Cat No 2962, Cell Signaling), p-MAPK (1:1000, Cat No 4370, Cell Signaling), MAPK (1:1000, Cat No 9122, Cell Signaling), P38 MAPK (1:1000, Cat No 9212, Cell Signaling), p-P38 MAPK (1:1000, Cat No 4511, Cell Signaling), p-mTOR (1:1000, Cat No 2971, Cell Signaling), mTOR (1:1000, Cat No 2972, Cell Signaling), p-RPS6/S6 ribosomal protein (1:1000, Cat No 2215, Cell Signaling), RPS6/S6 ribosomal protein (1:1000, Cat No 2217, Cell Signaling), CD133 (1:1000, Cat No 5860, Cell Signaling), SOX2 (1:1000, Cat No 3579, Cell Signaling), ALDH (1:1000, Cat No 36671, Cell Signaling). The β-actin antibody was from Sigma-Aldrich (1:5000, A2066).

### Generation of CDDP-resistant and radiation-resistant neuroblastoma cells

BE(2)-C and SK-N-AS (3 × 10^5^) cells were plated into 100 mm × 20 mm plates and cultured as described above. After incubation for 24 h, cisplatin was added at a final concentration of 50% survival dose (5 μM) and a ^137^Cs irradiator was irradiated with 50% survival dose (10 Gy) of radiation dose (J. L. Shepherd and Associates, Glendale, CA, USA) at room temperature (dose rate 1.8 Gy/min). After incubation for 7 days, the media was removed, and surviving cells were allowed to recover for a further 25 days. This development period was carried out for approximately 6 months, the 50% survival doses, 5 uM of CDDP and 10 Gy of radiation were re-assessed in each resistant cell line.

### Cell proliferation analysis

Cells (5000 cells/well) were seeded onto 96-well plates in RPMI 1640 culture media containing 10% FBS and grown for up to 4 days. Cell viability was assessed using CCK-8 kit daily. The values corresponding to the number of viable cells were read at OD450 with the FlexStation 3 Microplate Reader (Molecular Devices, Sunnyvale, CA, USA).

### Endothelial cell tube formation assay

HUVECs grown to ~70% confluence were trypsinized, counted, and seeded with various conditioned media at 48,000 cells per well in 24-well plates coated with 300 μl of Matrigel (BD Biosciences, Cat No. 354234). These cells were periodically observed by microscope as they differentiated into capillary-like tubule structures. After 6 h, cells were stained with Hematoxylin & Eosin and photographs were taken via microscope. The average number of tubules was calculated from an examination of three separate microscopic fields (200×) and representative photographs were obtained.

### Immunoblotting

Cells (5 × 10^5^) were collected at various time points and then washed with ice-cold PBS twice before adding lysis buffer (M-PER Mammalian Protein Extraction reagent, Thermo Scientific, Cat No. 78501) and cocktail inhibitor (Sigma, 5 μg/ml, Cat No. P8340). Equal amounts of protein were loaded into each well and separated by NuPAGE 4–12% Bis-Tris gel, followed by transfer onto PVDF-membranes (Bio-Rad, Cat No. 162-0177). Membranes were blocked with 5% non-fat milk in PBS-T for 1 h at room temperature. The blots were then incubated with antibodies against GRPR, Caspase-3, p-AKT1, AKT1, p-AKT2, AKT2, p-mTOR, mTOR, p-MAPK, MAPK, p-RPS6, RPS6, p-p38 MAPK, p38 MAPK CD133, Sox2, and ALDH for 1 h at 4°C. Goat anti-rabbit IgG secondary (1:5000; Santa Cruz Biotechnology, Cat No. sc-2004) was then incubated for 45 min at room temperature. Immunoblots were developed by using the chemiluminescence detection system (PerkinElmer, Cat No. NEL105) and autoradiography was performed. β-actin was used as a loading control.

### *In vitro* scratch assay

To measure cell migration *in vitro*, a confluent monolayer of cells in a 6-well plate was scraped with a 200 μl pipet tip and was incubated and observed microscopically at 24 to 72 h. Wound closure was calculated by measuring the remaining space in the microscopic images.

### Sphere cell culture and quantification of tumorsphere formation

For sphere formation, parent BE(2)-C, CDDP-R BE(2)-C, Rad-R BE(2)-C, parent SK-N-AS, CDDP-R SK-N-AS, and Rad-R SK-N-AS cells were cultured in a 1:1 mixture of DMEM and Ham’s F12, with B27 supplement (1×), epidermal growth factor (20 ng/ml) and fibroblast growth factor (40 ng/ml) on polystyrene-coated, ultra-low attachment plates (Corning Inc., Corning, NY, USA). Following 5 days in culture, spheres containing over 10 cells were quantitated by inverted phase contrast microscopy (Olympus CK40, Tokyo, Japan).

For quantification of tumorsphere formation, extreme limiting dilution analysis (ELDA) was performed with DMSO, CCT128930 (10 μM), PD98059 (100 μM), or both CCT128930 (10 μM) and PD98059 (100 μM). Several dilutions were serially completed on ultra-low attachment 96-well plates (Corning Inc., Corning, NY, USA) according to final cell doses of 500, 250, 125, 62, 31, 16, 8, and 4 cells/well (6 wells/group) in 100 μl of sphere media as described above. After incubation for 4 days, each well was quantified as “positive” or “negative” for SFF, determined by the presence of ≥1 sphere consisting of ≥10 cells. The statistical analysis of data from limiting dilution assay was performed at online using the soft program of the Walter and Eliza Hall, Institute of Medical Research [37].

### Statistical analysis

All results are shown as the mean value ± SEM from at least three independent experiments. Immunoblot scans are representative of three independent experiments. Statistical analysis was performed with Student’s *t*-test and *p* value < 0.05 was considered to be statistically significant.

## SUPPLEMENTARY MATERIALS



## Abbreviations

CCK-8: Cell Counting Kit-8
CDDP: cisplatin
CSCs: cancer stem cells
HUVECs: human umbilical vein endothelial cells
MAPK: mitogen-activated protein kinase
NSCLC: non-small cell lung cancer
SFF: sphere forming frequency
