# Supplementary material for: Dual-Targeting AKT2 and ERK in cancer stem-like cells in neuroblastoma
Source: Oncotarget. 2019 Sep 24;10(54):5645–59. doi: 10.18632/oncotarget.27210 (PMC6771463; doi:10.18632/oncotarget.27210)
Supplement: Supplementary file 1 [file oncotarget-10-5645-s001.pdf]

Dual-Targeting AKT2 and ERK in cancer stem-like cells in neuroblastoma

SUPPLEMENTARY MATERIALS

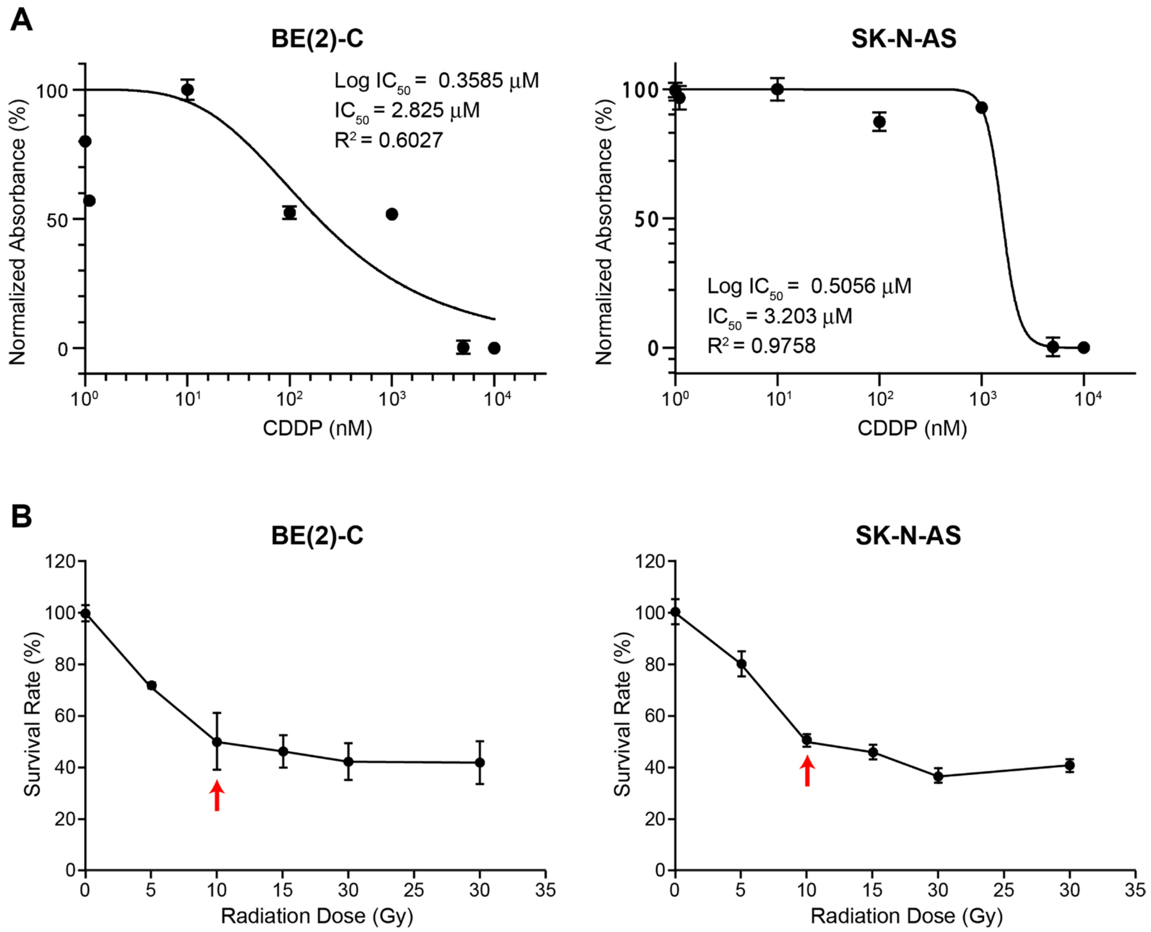

Supplementary Figure 1: Effect of CDDP (IC_{50}) and Radiation (50% of cell survival inhibition) doses on viability of neuroblastoma cells. (**A**) BE(2)-C and SK-N-AS cells were treated with increasing concentrations of cisplatin (CDDP, 1 nM–10,000 nM) for 96 h. Cell survival was measured using Cell Counting Kit-8. IC50 concentrations were assessed for each cell line using dose-response curves generated by GraphPad Prism 8 software. (**B**) Effect of radiation dose on the viability of BE(2)-C and SK-N-AS cells.

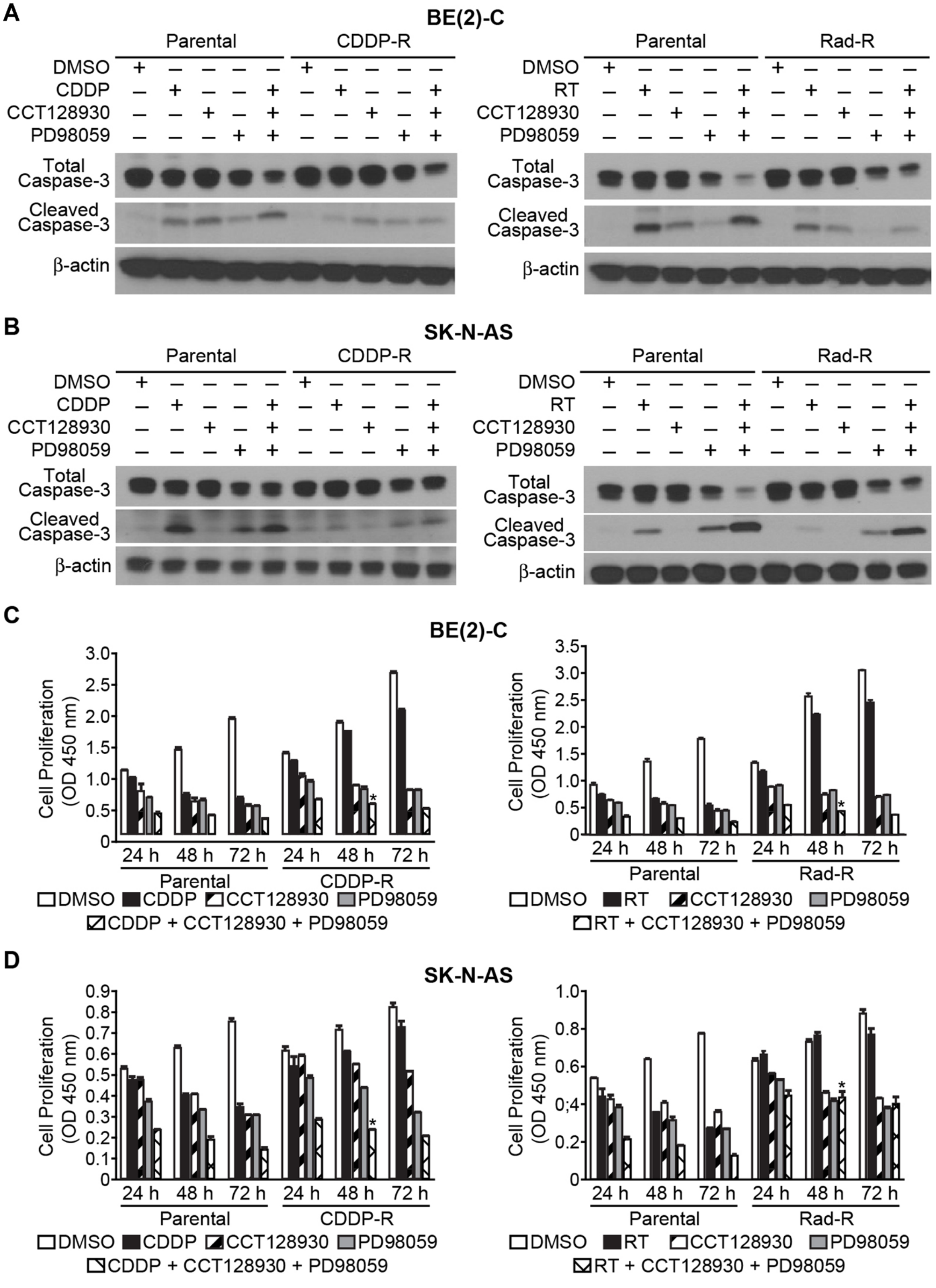

Supplementary Figure 2: Effect on proliferation and apoptosis of combination treatment of CDDP, CCT128930, and PD98059 in CDDP-R/Rad-R human neuroblastoma cells. (**A**) Cleaved caspased-3 was inhibited in the CDDP-R and Rad-R BE(2)-C cells when compared with parental BE(2)-C cells treated with combination groups of both CCT128930 (10 μM) and PD98059 (100 μM) after treatment with CDDP (5 μM) for 48 h. (**B**) Cleaved caspased-3 was inhibited in CDDP-R and Rad-R SK-N-AS cells treated with combination groups of both CCT128930 (10 μM) and PD98059 (100 μM) when compared with parental BE(2)-C cells/SK-N-AS cells treated with combination groups of both CCT128930 (10 μM) and PD98059 (100 μM) after irritation with ^{137}Cs (10 Gy) for 48 h. (**C**) Proliferation was inhibited in CDDP-R and Rad-R BE(2)-C cells treated with both CCT128930 (10 μM) and PD98059 (100 μM) when compared with parental BE(2)-C cells treated with combination groups of both CCT128930 (10 μM) and PD98059 (100 μM) after treatment with CDDP (5 μM) for 48 h. Data are the mean ± SEM. * Significantly different at $p < 0.005$ vs. Parental. (**D**) Proliferation was inhibited in CDDP-R and Rad-R SK-N-AS cells treated with both CCT128930 (10 μM) and PD98059 (100 μM) compared with parental SK-N-AS cells treated with combination groups of both CCT128930 (10 μM) and PD98059 (100 μM) after irritation with ^{137}Cs (10 Gy) for 48 h. Data are the mean ± SEM. * Significantly different at $p < 0.005$ vs. parental.

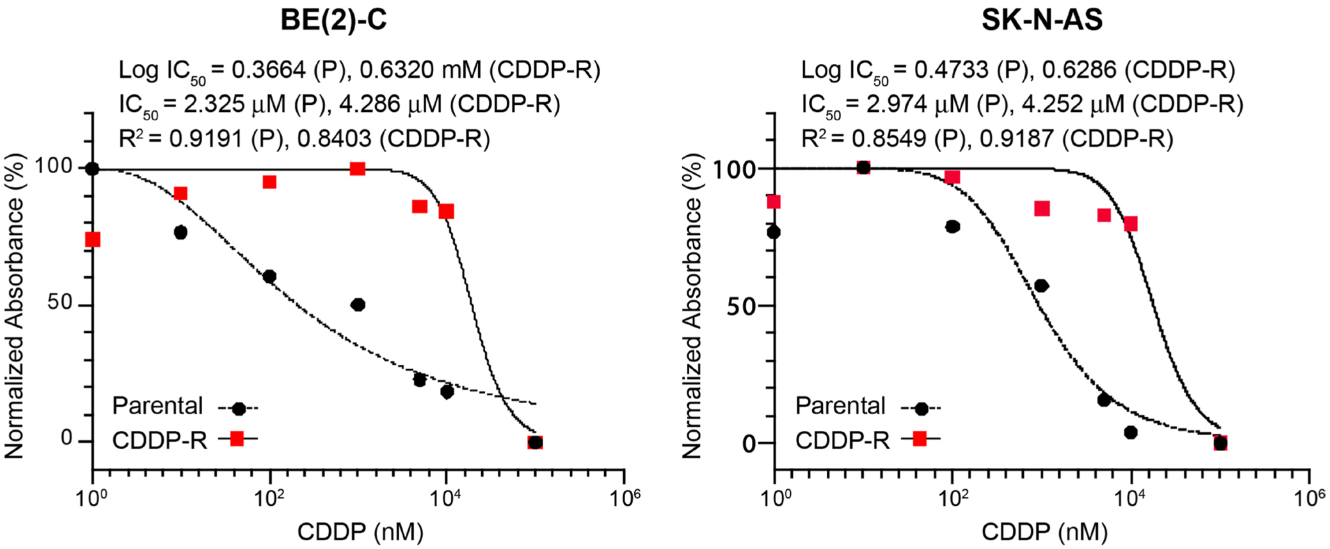

Supplementary Figure 3: CDDP-R human neuroblastoma cells display increased changes at IC_{50} concentration. CDDP-R BE(2)-C cells/CDDP-R SK-N-AS cells increase IC_{50} concentrations according to concentration of cisplatin (10 nM–100,000 nM) for 72 h. IC_{50} concentrations were re-assessed for each cell line using dose-response curves generated by GraphPad Prism 8 software. A significant increase in IC_{50} concentration was determined for each CDDP-R cells line compared to parental cell line.

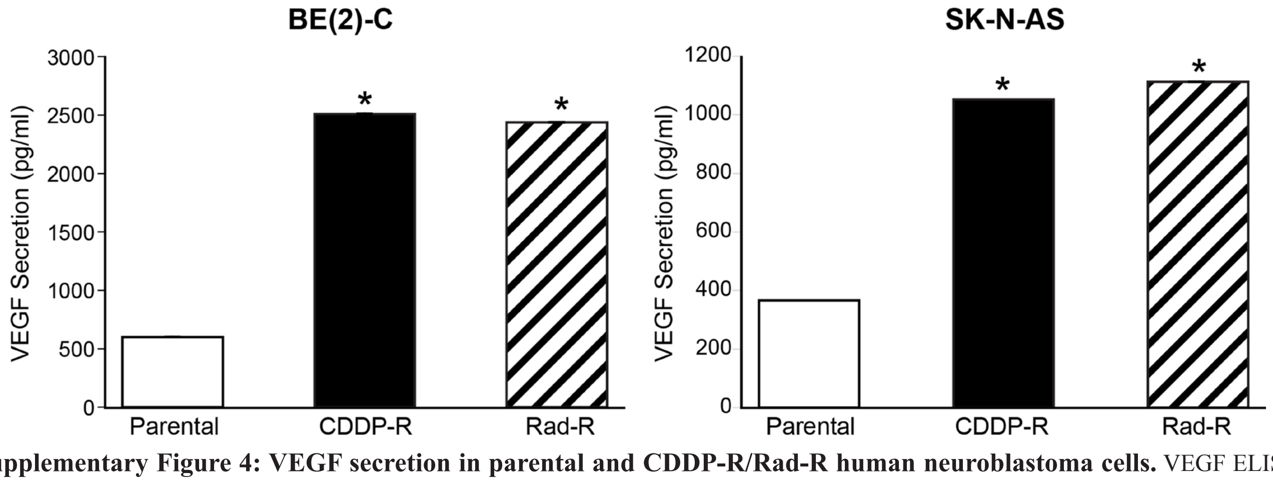

Supplementary Figure 4: VEGF secretion in parental and CDDP-R/Rad-R human neuroblastoma cells. VEGF ELISA was performed with cell culture supernatant used for HUVECs tubule formation in Figure 3 E. ELISA was completed in triplicate (means ± SEM; * = $p < 0.05$ vs. Parental).
